# Non-Suicidal Self-Injury and Depressive Symptoms During Adolescence: Testing Directionality

**DOI:** 10.1007/s10964-025-02183-y

**Published:** 2025-04-13

**Authors:** Lauree Tilton-Weaver, Rebecca Schwartz-Mette

**Affiliations:** 1 Örebro University, Örebro, Sweden; 2 State University of New York at Buffalo, Buffalo, USA

**Keywords:** Non-suicidal self-injury, Depressive symptoms, Adolescence, Transactional, Bidirectional

## Abstract

As risk for both non-suicidal self-injury and depression increases at adolescence, it is critically important to clarify equivocal findings regarding the temporal ordering of these health concerns. Addressing the limitations of past research, which included the use of only two data waves and analyses that do not account for within-person variance, the aim in this study was to examine the temporal ordering of non-suicidal self-injury and depressive symptoms during adolescence. Two independent samples were examined. One sample (first used by Marshall et al., 2013) consisted of 799 Swedish adolescents (11 to 15 years, *M*_*age*_ = 13.18, *SD* = 0.74; 51% girls). The second sample was a later cohort of adolescents from the same area (*N* = 2760; aged 12 to 18 years, *M*_age_ = 13.75, *SD* = 0.74; 49% girls). Analyses were two-part cross-lagged panel models (CLPMs) and random-intercept cross-lagged panel models (RI-CLPMs) to account for the semi-continuous distribution of NSSI and to determine if traditional lagged models were adequate for estimating within-person temporal ties. The results suggest that traditional CLPMs had inadequate fits to data. RI-CLPMs showed significant within-person, transactional associations between depressive symptoms and NSSI. Specifically, NSSI at a mean age around 13 years was associated with increases in depressive symptoms one year later, which were in turn associated with increases in NSSI when the average age was about 15 years old. Subsequently, lags suggested the potential for maintenance of comorbid distress. These results suggest that efforts to reduce depression and NSSI during adolescence need to begin in early adolescence, with a focus on adaptive responses to distressing emotional experiences.

## Introduction

Adolescence is a formative period involving heightened sensitivity to risk factors implicated in the development of psychopathology. During adolescence, rates of non-suicidal self-injury (NSSI) and depressive symptoms rise (e.g., Shorey et al., 2022), underscoring the importance of understanding the etiology and timing of these health-risk concerns. Indeed, researchers and practitioners have noted that NSSI and depressive symptoms co-occur in adolescence (e.g., Niu et al., 2024), leading researchers to ask whether these two mental health issues co-develop or if one issue temporally precedes the other. Past studies have initially examined this question, but were limited to only a few waves (e.g., two) or by analyses that tend yield estimates that cannot capture individual-level change. In this study, the aim was to extend research by addressing questions about temporal ordering with more comprehensive data across a wide adolescent age range and more rigorous analytical methods.

### Non-Suicidal Self-Injury and Depressive Symptoms in Adolescence

Researchers conceptualize non-suicidal self-injury (NSSI) as deliberate injury to or destruction of one’s own body tissue(s) in the absence of suicidal intent (Muehlenkamp & Gutierrez, 2004). NSSI may take many forms, including but not limited to hitting, burning, piercing, cutting, and pulling hair (Nock, 2008). Many individuals who struggle with NSSI may use multiple methods (Klonsky, 2011). Meta-analyses suggest that peak age of onset for NSSI is in early to middle adolescence (De Luca et al., 2023) with highest observed prevalence rates in adolescence (17.2%; Swannell et al., 2014) as compared to earlier or later developmental stages (e.g., Plener et al., 2015). Trajectory analyses suggest a peak in NSSI around age 15, decreasing thereafter (Barrocas et al., 2012). This pattern appears to hold for many, but not all adolescents (Tilton-Weaver et al., 2023).

According to both theory and empirical research, NSSI serves multiple regulatory functions that can be understood through a biobehavioral lens. Biological models suggest that NSSI is correlated with challenges regulating emotions and an attenuated pain response (Kaess 2022), and the Four Function Model (Nock & Prinstein, 2004) posits that NSSI is maintained through automatic / intrapersonal and interpersonal contingencies (i.e., NSSI is reinforced within the self or by others) as well as by positive and negative types of reinforcement (i.e., introduction of a favorable or removal of a negative stimulus). Negative affect often precedes and is reduced following NSSI (Kuehn et al., 2022), and individuals who engage in NSSI most frequently cite interpersonal and emotion regulatory functions for their self-injurious behavior (Taylor et al., 2018).

Depressive symptoms are even more common during adolescence, with approximately 34% of adolescents endorsing impactful depressive symptoms (Shorey et al., 2022) and nearly 16% who qualify for a diagnosis of depressive disorder (Daly, 2022). These rates reflect a relative increase in depressive symptoms in adolescence as compared to childhood, and rates stay elevated into early adulthood before declining in later life (Natsuaki et al. 2009). Symptoms of depression include negative affect (e.g., sadness, irritability), loss of interest and pleasure in daily activities, appetite and sleep difficulties, feelings of guilt and worthlessness, and thoughts of suicide (American Psychiatric Association, 2013).

### Links Between NSSI and Depressive Symptoms

NSSI and depressive symptoms can co-occur during adolescence and particularly among those adolescents with more severe symptomology (Tilton-Weaver et al., 2019). In clinical reports, up to 76% of adolescents who experienced depression also engaged in NSSI (Zhang et al., 2023), and among adolescent psychiatric outpatients who self-injured, almost 95% were depressed (Niu et al., 2024). Such co-occurrence can be explained by one problem contributing to the other. The prevailing question is which problem comes first?

One hypothesis is that symptoms of depression, which include negative emotions and numbness, precede NSSI (Nock, 2008), as NSSI serves as a way of coping with such feelings (Klonsky et al., 2013). This direction is empirically supported: researchers have found that symptoms of depression predict later NSSI (e.g., Barrocas et al., 2015) and mediate linkages between psychosocial risk factors and NSSI (e.g., parent rejection; Zhu et al., 2020; peer problems, Wei et al., 2021; victimization, Liu et al., 2023). Likewise, depression has been identified as one of the strongest risk factors for NSSI in meta-analyses (Fox et al., 2015).

As for the opposite causal direction, it is plausible that NSSI precedes depressive symptoms. Although NSSI reduces negative emotions and emotions in the short-term, relief brought on by NSSI is temporary. In addition, individuals who have engaged in NSSI report experiencing shame, guilt, and self-directed anger (Klonsky et al., 2013). Shame, guilt, and negative self-evaluations are theoretical risk factors for depressive symptoms (Rudolph et al., 2008). Also, engaging in NSSI is stigmatized by others (particularly when signs of scarring are evident), including by parent, peers, and teachers (Lloyd et al., 2018). Adolescents who have engaged in NSSI experience peer victimization, loneliness, and other relationship difficulties (De Luca et al., 2022). Collectively, these consequences of NSSI could lead to increased symptoms of depression.

In addition, both directions of causality may operate, but perhaps at different time frames. Studies of the onset of NSSI and depression suggest that both are rarely seen in childhood but rise during adolescence (NSSI: Liu et al., 2022; depression: Avenevoli et al., 2008). Estimates from Dutch (Gandhi et al., 2018) and Norwegian samples (Tørmoen et al, 2020) suggest that the age of onset for NSSI peaks between 14 and 15 years of age. By comparison, the age of onset for depression is estimated as much later (~age 17 years) because diagnoses of depressive disorders are used. However, increases in depressive symptoms are seen in early adolescence (Maughan et al., 2013), especially among girls. Such estimates coincide with trajectory analyses of depression symptoms suggesting increases slightly earlier than NSSI (Kwong et al., 2019). In summary, previous research suggests that depressive symptoms may precede NSSI temporally but operate bidirectionally later in adolescence.

### Empirical Evidence of Directionality

Robust evidence connecting depression and NSSI underscores the importance of examining the directionality of ties to best understand their development over time. To date, only seven published studies have simultaneously examined both directions, using lagged models. They differ in the age ranges of their samples, computation of NSSI, and in lag duration (Supplemental Tables 1 and 2 provide more specific information about these studies).

Of the three studies using only two waves of data, two found evidence of bidirectionality (Hu et al., 2024; Faura-Garcia et al., 2024), with testing and support that the lag from depressive symptoms to NSSI was stronger than the opposite direction (Hu et al., 2024). The third study found a significant lag only from depressive symptoms to NSSI (Garisch & Wilson, 2015).

Three studies incorporated more than two measurement occasions. Leveraging three or more waves of assessment allows for examining transactional patterns (e.g., depressive symptoms associated with changes in NSSI and subsequent changes in depressive symptoms). In addition, analyzing three or more waves provides a means of examining whether a particular direction of effect is limited to a specific age range or is consistent, and thus robust, over time. Of these four studies, the earliest study (Marshall et al., 2013) only found one significant path, from depressive symptoms to NSSI in the first lag (when the adolescents were aged 12 to 14). In the other studies (Liu et al., 2024 and Buelens et al., 2019, which aggregated symptoms of depression with anxiety), evidence supported bidirectionality across both lags.

Related evidence comes from a study examining deliberate self-harm using RI-CLPM to three annual waves of data (Liu et al., 2024). In this study, the CLPM was a better fit to the data. The results suggested bidirectional associations during both lags, spanning the ages of 10 to 16 years at T1 to 12 to 18 at T3. However, because the measure used was deliberate self-harm, which can include suicidal behaviors, we are not able to compare this study with the others.

In summary, most studies have found evidence to support the idea that NSSI and depressive symptoms are bidirectionally related. Despite this important initial work, questions remain that preclude firm conclusions from being drawn. These issues include the analytic approach chosen and the measurement of NSSI.

### Distinguishing Individual-level Processes From Between-Person Differences

The analytical choice for all of the studies was cross-lagged panel models (CLPM), which has been used to assess transactional associations. Although the CLPM accounts for temporal stability by estimating autoregressive parameters, it assumes that individuals in the dataset vary over time around a common (i.e., group) mean and does not address the plausibility of trait-like stable, individual differences over time (Lucas, 2023). In other words, although measurement occasions are technically nested within individuals in longitudinal data, there is no separation of variance at the within-person level from the between-person level. If the stability of individual differences for a given construct is reasonably high, as is often the case in psychological research, autoregressive parameters within the CLPM will not adequately control for this. Thus, resultant estimates may be biased, precluding clarity and accuracy of estimated directions of effect (e.g., Finkel, 1995).

Statisticians proposed the RI-CLPM as an extension of the CLPM that accounts for both temporal stability as well as time-invariant, trait-like stability within people (Hamaker et al., 2015). The RI-CLPM includes a random intercept to separate within-person processes from between-person differences. In the RI-CLPM, an individual’s temporal deviations are calculated from their expected individual mean scores, not the group’s means. Applied to our study, using RI-CLPM to test transactional associations of NSSI and depressive symptoms allows for clarification of whether associations between them, as have been observed in the literature, are reflective of associations within the same adolescents or, rather, of associations between them.

In the sole study testing if between-person variance needed to be estimated (Liu et al., 2024), the results suggested that the CLPM was sufficient. However, the estimates from this study used a measure of deliberate self-harm, which is not equivalent to NSSI. Thus, the question of whether the estimates of previous studies are relevant remains unanswered.

### NSSI: A Semi-Continuous Variable

Scholars have struggled with how to capture variation in NSSI. Many chose to use binary assessments, asking if youth have or have not engaged in NSSI, as was done in two of the existing studies (Buelens et al., 2019; Hu et al., 2024). Both of these studies also assessed lifetime engagement, rather than limiting the timeframe to match the duration of the lags. The remaining studies used continuous measurement, ranging from no engagement (0) to more frequent engagement. Neither treatment fully captures the distribution of NSSI, which is best described as semi-continuous (with zero-inflated distributions). Semi-continuous variables are distributed such that there are many zero values (i.e., zero-inflated distributions), and non-zero values are highly skewed. They are common in psychological research but are rarely treated in a way that reflects their complexity.

With respect to NSSI, in community samples the majority of adolescents report no engagement in NSSI, with many zero values. The remainder of the distribution is positively skewed, with frequencies dropping as levels of NSSI increase. None of the studies reviewed address this issue, modeling either the binary component or ignoring high number of youth reporting no NSSI. Modeling the components separately does not solve the problem, as this tends to bias the estimates by failing to account for associations between the binary and continuous elements (e.g., individuals who engage in NSSI tend to do so at low levels). This makes the results of previous studies tentative, creating a gap that needs to be addressed with appropriate analyses.

### Gender As a Moderator

Despite new meta-analytic evidence that girls report engaging in more NSSI than do boys in many regions of the world, including Europe (Moloney et al., 2024), studies examining links of NSSI and depressive symptoms over three or more waves of data have not observed significant gender differences. However, gender differences are well-established for depressive symptoms and have also been found in research on NSSI. Specifically, girls tend to report higher levels of both depressive symptoms (Shorey et al., 2022) and NSSI (Moloney et al., 2024). Although mean-level differences are not always the same as process differences, there are theoretical and empirical reasons to believe that the linkages between NSSI and depressive symptoms may be more applicable to girls than to boys. For example, theoretical frameworks for depressive symptoms in girls focus on the processes that may be specific to girls’ depressive symptoms to NSSI, such as sensitivity to negative emotions, negative self-construals, and relationship stressors (Rudolph et al., 2008). Thus, gender differences should continue to be examined.

## The Current Study

Although existing research indicates that NSSI and depressive symptoms are related bidirectionally during adolescence, there is reason to treat the evidence as tentative. Specifically, the methods and analyses used in previous work may have resulted in biased information, such that replication is needed. In this study, the replicability of previous work was tested by applying recently advanced statistical techniques to longitudinal data in two independent samples. In early adolescence, depressive symptoms were expected to be related to increases in NSSI, but not the reverse. After early adolescence, positive and bidirectional associations between NSSI and depressive symptoms were expected. No firm hypotheses were put forward about differences in magnitude of bidirectional lags within timeframes or for differences in magnitude of the same paths over time. As gender moderation was exploratory, no firm hypotheses were put forward.

## Method

### Sample 1

#### Sample and procedures

The first sample came from the same cohort-sequential study used by Marshall & colleagues (2013), the *7 Schools Study*. The participants were 7^th^ to 9^th^ grade students from 7 selected middle schools in one urban area of central Sweden. The community was similar to the rest of Sweden in terms of income, migrant composition, parent education, and unemployment. In the initial waves of data collection (2009), all students were targeted (1555 participated, 91% response). Additional data collections were conducted annually. This study used waves 1 to 4, one more wave of data than that used by Marshall et al. (2013).

The analytic sample included only cases for whom scales could be calculated in at least two of the three waves. The sample ranged in age from 11 to 15 years at T1 (*M* = 13.18, SD = 0.74), of whom 51% were girls. The majority were born in Sweden (90%), with Swedish-born mothers (74%) and fathers (72%). Most reported intact families (62%) and living with both parents (67% lived with both parents, some of whom were never married; an additional 16% lived in joint-custody arrangements).

Binary logistic regressions, comparing those who were retained in the analytic sample from those excluded, examined potential differences in age, gender, birthplace, parent birthplace, parent education, and indicators of SES (i.e., disposable income). The analyses indicated that adolescents who were eliminated from analyses were older than those retained at T1 and T2, but younger at T3 and T4. At T3 and T4, they were also more likely to be the children of non-Swedish parents (mothers only at T3). No other significant differences were found, suggesting that cases eliminated may have contributed to some reduction in representativeness, but were unlikely to contribute to Type II error.

### Measures

#### NSSI

In the original Marshall et al. (2013) study, NSSI was assessed using the shortened version of the Deliberate Self-Harm Inventory (Lundh et al., 2007; original in Swedish) with a stem added to prompt reporting of self-harm that did not entail a desire to die (Tilton-Weaver et al., 2019). Adolescents reported how many times in the previous 6 months they had inflicted self-injuries without suicidal intent. NSSI scores were calculated as the mean of 9 forms of self-injury (e.g., cutting, burning), with responses ranging from 0 (none) to 5 (five or more times). Cronbach alphas indicated good reliability: α_t1_ = 0.82, α_t2_ = 0.91; α_t3_ = 0.92; α_t4_ = 0.93.

#### Depressive symptoms

A Swedish language version of the Center for Epidemiological Studies-Depression for Children scale (CES-DC; translation in Olsson & Von Knotting, 1997) was used to assess depressive symptoms. As to think about the past week, students recorded the frequency of depressive symptoms for 16 items (e.g., “I felt down and unhappy,” “I was bothered by things that usually don’t bother me”), using a response scale ranging from 1 (“not at all”) to 4 (“often”). Note that this shortened version of the CES_DC does not use the positively worded items (“I feel happy”) because they reduce the internal consistency. The mean of all items served as scores. Cronbach alphas indicated very good reliability: α_t1_ = 0.93, α_t2_ = 0.93, α_t3_ = 0.94, α_t4_ = 0.93.

#### Procedures

Prior to data collection, the responsible researchers obtained approval from the regional ethics board for all procedures and measures. Parents were informed of the study’s purposes and procedures, with the opportunity to withdraw consent for their adolescents’ participation via prepaid postcards, email, and telephone. Only 1% declined. Students were then informed about the study and their rights (to confidentiality, refusal to answer questions, the ability to withdraw from the study at any point without repercussions). Only 3% declined to participate. After establishing consent, trained research assistants collected data via questionnaires, during classes, with teachers excused from the rooms. Students were provided with refreshments during a mid-point break and given a small gift at the end.

#### Missing data

Across all four waves, 18% of the scale measurements could not be calculated due to missing data. Little’s test suggested that these data could be treated as missing at random (MCAR), *X*^2^ (52) = 61.49, *p* = 0.17.

### Sample 2

#### Sample and procedures

The data for the second sample comes from the *3 Cities Study*, a cohort-sequential study of mental health issues, using the same procedures used for the *7 Schools Study*. In three municipalities in central Sweden (one of which was the same as the *7 Schools Study*), students in all 17 middle schools were targeted. Participants were in 7^th^ and 8^th^ grade at the first data collection (2013). Annual data collections continue for another 4 years, during the spring term (roughly from the beginning of February to the beginning of June).

As with the *7 Schools Study*, approval for the procedures and measures was obtained from the regional ethics board before collecting data. Annually, data collection proceeded as follows. Parents were mailed information about the study and given the same opportunity to deny consent for their adolescents. Less than half a percent denied consent. Data collection by trained research assistants took place at the schools, during school hours, with teachers absent. Students were informed of the study and their rights, then provided with questionnaires. During the study, they were given refreshments and a break in the middle of data collection. Each class was given a 300 SEK for their class trips (~30 Euros). Each year, assistants returned to collect data from students who were still enrolled and any additional students who wanted to participate.

Each year, all students in the appropriate grades were targeted. Target sample sizes were as follows: *n*_*t1*_ = 3336, *n*_*t2*_ = 3352, *n*_*t3*_ = 4038, *n*_*t4*_ = 4049, *n*_*t5*_ = 3782. Response rates within each wave ranged from 75% to 88%. Retention rates across waves ranged from 91% between T1 and T2 to 41% for all five waves.

Of the sample of adolescents who participated in at least one wave (*n* = 4814, 51% boys), we selected adolescents who had participated in at least 3 of the 5 assessments. This results in an analytic sample of 2760 adolescents. The analytic sample ranged in age from 12 to 18 years at T1 (*M* = 13.75, SD = 0.74), with 49% girls and 51% boys. Demographically, majority were born in Sweden (89%). Their mothers and fathers were primarily born in Sweden (75%, 74%), or in other Nordic countries (2%). Other mothers and fathers were born in other European countries (6%) or outside of Europe (17%). The majority reported that their parents had not divorced or separated during the course of the study (65%). Most reported living with both parents (66%), in shared custody arrangements (22%), with either the mother or the father (9 and 2%), or with someone else (1%). The measures of disposable income, tapping families’ economic situations, showed that most participants’ families owned at least one car (94%) and multiple computers (93%). Most reported having their own bedroom (90%) and income to go on at least one family vacation per year (87%). These indicators suggest that few families were impoverished, as is consistent with economic indicators in the region from which this sample was drawn.

Five binary logistic regressions compared adolescents who were retained for analyses to those excluded (one for each wave), using age, gender, demographic variables, and the scores for NSSI and depressive symptoms at that wave as predictors. Across these analyses, adolescents who were excluded were more likely to be: older (T1, T2, T3) and born outside of Sweden (T3, T4, T5), living in a home with only one parent (T1, T3, T4, T5) and in a home where a language other than Swedish was spoken (T4) than those who were retained. Compared to cases retained, those excluded also reported having less disposable income in the family, as indicated by sharing a bedroom (T2), taking fewer vacations (T2), or having fewer computers at home (T4 and T5). In addition, excluded adolescents reported higher levels of NSSI (T2, T3, T4) and depressive symptoms (T2). Thus, excluding those with fewer waves likely reduced variability in ethnic background, economic disparities, and in the modeled variables. This may have contributed to higher Type II error rates.

#### Measures

The same measures that were used in *7 Schools* was used in this study, with one minor alteration. At T1, the scaling for the Swedish version of the CES-DC was inadvertently changed to 5 (1 = not at all; 5 = often). The scaling was changed back to the original 4-point scale at T2. Although the positively worded items were available for the CES-DC, we chose to omit them, for consistency with Sample 1.

Cronbach alphas for the DSHI, assessing NSSI indicated good to excellent reliability (α_t1_ = 0.89, α_t2_ = 0.86, α_t3_ = 0.88, α_t4_ = 0.86, α_t5_ = 0.85). Similarly good reliability was found for the CES-DC, assessing depressive symptoms (α_t1_ =0.94, α_t2_ = 0.92, α_t3_ = 0.91, α_t4_ = 0.93, α_t5_ = 0.93).

#### Missing data

Across all five waves, 19% cases had missing data that precluded scale calculations. Little’s test suggested that these data could be treated as missing at random (MCAR), *X*^2^ (238) = 222.56, *p* = 0.76.

### Plan of Analysis

SPSS (v. 28) was used for data management and calculation of descriptive statistics and correlations. Due to high levels of skew for NSSI, all correlations involving NSSI were Spearman’s Rho. Directionality of associations between NSSI and depressive symptoms was tested in *Mplus* (v. 8.7; Muthén & Muthén, 1998–2023). As NSSI distributions were semi-continuous, the CLPM and RI-CLPMs were two-part models, using Bayesian estimation, following procedures provided by Muthen et al (2024) for model commands, assessments of model fit, and significance testing of path estimates (commands are provided in an online supplementary file). This procedure entails *Mplus* creating two components (parts): a binary variable (0 = no NSSI; 1 = any NSSI) and a continuous variable (0 is treated as missing, all non-zero values are retained). The components are then modeled using Bayesian estimation with full-information maximum likelihood estimation (FIML) to handle missing data. Modeling includes estimating random intercepts, within-time associations between variables, associations over time involving the same variables, and estimating the cross-lagged paths between the variables of interest.

As described by Muthén et al. (2024), fit was evaluated by examining the Positive Predictive P-value (PPM), where values of 0.05 or lower indicate poor fit and values closer to 0.5 indicate excellent fit. Significance testing entailed calculating credibility intervals (akin to confidence intervals), using the posterior standard deviation. The 95% credibility intervals were used, with significance indicated when the credibility intervals did not contain zero. Where applicable, the relative strength of cross-lagged paths was tested, to examine strength of bidirectionality (i.e., comparisons across variables within the same lag) or to examine strength over time (i.e., comparisons of the same variable across different lags). Compared paths were constrained and then freely estimated, using the Wald’s χ2 difference test.

Moderation by gender was tested using procedures described by Muthen et al. (2024), with mixture modeling using known classes. The change in fit was evaluated by examining the change in PPP, using more constraints to test individual estimates when needed. *MPlus* commands for all analyses can be found in the online supplemental file.

## Results

### Preliminary Analyses

Descriptive statistics and correlations between study variables are reported in Tables 1 and 2. On average, rates of NSSI and depressive symptoms were low. As expected, NSSI and depressive symptoms were positively and significantly correlated.

**Table 1:** Descriptive Statistics for Study Variables

	Sample 1 (*M*_*age*_ = 13.18, *SD* = 0.74)		Sample 2 (*M*_*age*_ = 13.75, *SD* = 0.74)	
Variable	*M*	*SD*	*M*	*SD*
Non-suicidal self-injury				
T1	0.20	0.53	0.12	0.43
T2	0.28	0.71	0.14	0.45
T3	0.25	0.78	0.14	0.52
T4	0.23	0.82	0.13	0.46
T5	–	–	0.13	0.46
Depressive symptoms				
T1	1.61	0.58	1.79	0.74
T2	1.71	0.61	1.70	0.60
T3	1.71	0.65	1.76	0.56
T4	1.63	0.60	1.84	0.65
T5	–	–	1.90	0.66

**Table 2:** Correlations Among Study Variables

	Variable	1	2	3	4	5	6	7	8	9
1	NSSI T1	–	0.48***	0.38***	0.27***	–	0.35***	0.26***	0.20***	0.21***
2	NSSI T2	0.43***	–	0.49***	0.39***	–	0.34***	0.46***	0.31***	0.25***
3	NSSI T3	0.33***	0.47***	–	0.48***	–	0.30***	0.28***	0.38***	0.39***
4	NSSI T4	0.29***	0.43***	0.46***	–	–	0.19**	0.16***	0.23***	0.29***
5	NSSI T5	0.22***	0.32***	0.37***	0.48**	–	–	–	–	–
6	Depressive symptoms T1	0.38***	0.30***	0.24***	0.23**	0.18***	–	0.58***	0.47***	0.29***
7	Depressive symptoms T2	0.28***	0.40***	0.29***	0.28**	0.19***	0.62***	–	0.49***	0.33***
8	Depressive symptoms T3	0.25***	0.30***	0.36***	0.30**	0.22***	0.52***	0.65***	–	0.41***
9	Depressive symptoms T4	0.24***	0.27***	0.26***	0.36**	0.24***	0.50***	0.56***	0.60***	–
10	Depressive symptoms T5	0.18***	0.23***	0.23***	0.26**	0.30***	0.44***	0.50***	0.56***	0.64***

Correlations for Sample 1 are above the diagonal; Sample 2 are below. Spearman’s rho is reported for all correlations involving NSSI.

**p < 0.01. ***p < 0.001.

Prior to modeling, longitudinal measurement invariance was tested. Scalar invariance over time was found for both samples for NSSI and depressive symptoms (additional information can be obtained from the 1^st^ author).

### Lagged Models, Sample 1

**Fig. 1:**
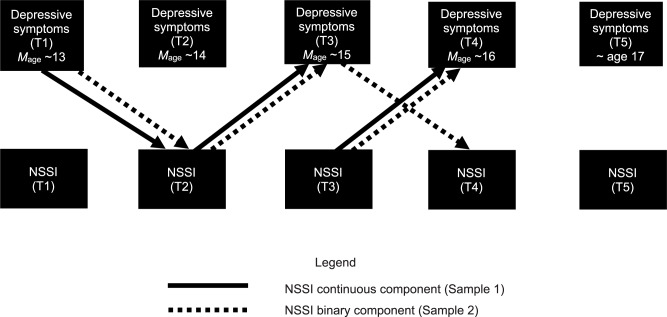
Significant cross-lagged paths from RI-CLPMs. NSSI continuous component (Sample 1). NSSI binary component (Sample 2)

Fit indices for the CLPM and RI-CLPM indicated that RI-CLPM was a better fit: the PPP for the CLPM was 0.092, with superior fit found for the RI-CLPM (PPP = 0.435). As can be seen in the left-hand columns of Table 3, the estimates that did not include 0 in the credibility intervals included the within-person lagged path from depressive symptoms at T1 to continuous levels of NSSI at T2, suggesting that depressive symptoms were related to increases in frequency of NSSI. Lagged within-person paths without 0 in the credibility intervals were also found in the opposite direction between T2 and T3 and between T3 and T4, from NSSI (continuous) depressive symptoms. These values were also positive, indicating that higher frequencies of NSSI at T3 and T4 were related to increases in depressive symptoms. These significant paths, all involving the continuous component of NSSI, are depicted with solid lines in Fig. 1.

**Table 3:** Estimates of Two-Part Model Paths for NSSI and Depressive Symptoms

	7 Schools (*M*_*age*_ = 13.18, *SD* = 0.74)				3 Cities (*M*_*age*_ = 13.75, *SD* = 0.74)			
		Posterior	95% Credibility Interval			Posterior	95% Credibility Interval	
Model path	*Est*.	*SD*	Lower	Upper	*Est*.	*SD*	Lower	Upper
Rank-order stability paths								
NSSI binary								
T1 to T2	0.06	0.14	−0.20	0.33	0.17	0.08	0.02	0.31
T2 to T3	0.23	0.18	−0.14	0.54	0.39	0.07	0.24	0.51
T3 to T4	0.38	0.17	0.01	0.66	0.43	0.06	0.30	0.54
T4 to T5	–	–	–	–	0.55	0.06	0.44	0.65
NSSI continuous								
T1 to T2	−0.10	0.26	−0.82	0.22	0.19	0.07	0.03	0.32
T2 to T3	0.42	0.08	0.24	0.5	0.33	0.06	0.20	0.44
T3 to T4	0.41	0.13	0.11	0.63	0.37	0.07	0.23	0.50
T4 to T5	–	–	–	–	0.55	0.06	0.43	0.65
Depressive symptoms								
T1 to T2	0.32	0.09	0.15	0.49	0.29	0.04	0.21	0.36
T2 to T3	0.09	0.08	−0.08	0.23	0.04	0.06	−0.08	0.14
T3 to T4	0.03	0.10	−0.18	0.20	0.02	0.05	−0.08	0.12
T4 to T5	–	–	–	–	0.31	0.03	0.24	0.37
Lagged paths								
NSSI binary to depressive symptoms								
T1 to T2	−0.01	0.09	−0.19	0.16	0.02	0.05	−0.08	0.12
T2 to T3	0.17	0.10	−0.03	0.37	0.18	0.07	0.05	0.31
T3 to T4	0.21	0.12	−0.02	0.42	0.19	0.06	0.08	0.30
T4 to T5	–	–	–	–	0.09	0.05	0.00	0.18
NSSI continuous to depressive symptoms								
T1 to T2	−0.15	0.13	−0.43	0.08	−0.02	0.05	−0.11	0.09
T2 to T3	0.16	0.08	0.01	0.31	0.08	0.07	−0.05	0.22
T3 to T4	0.31	0.11	0.10	0.51	0.03	0.07	−0.01	0.17
T4 to T5	–	–	–	–	0.03	0.05	−0.07	0.13
Depressive symptoms to NSSI binary								
T1 to T2	0.21	0.11	0.00	0.41	0.15	0.05	0.05	0.25
T2 to T3	0.04	0.12	−0.17	0.31	0.06	0.06	−0.05	0.17
T3 to T4	0.12	0.12	−0.11	0.36	0.11	0.05	0.001	0.21
T4 to T5	–	–	–	–	0.00	0.05	−0.09	0.09
Depressive symptoms to NSSI continuous								
T1 to T2	0.24	0.14	0.01	0.56	0.09	0.05	−0.16	0.19
T2 to T3	−0.02	0.07	−0.16	0.12	0.03	0.06	−0.09	0.1
T3 to T4	−0.01	0.12	−0.25	0.23	−0.01	0.07	−0.14	0.13
T4 to T5	–	–	–	–	0.10	0.05	−0.01	0.20

The magnitude of the lags from NSSI (continuous) to depressive symptoms varied between T2 to T3 and between T3 to T4. The test comparing them, however, was not significant (Wald’s χ2 difference = 0.39, *df* = 1, *p* = 0.53), suggesting similar magnitudes.

The model used to test gender differences had poor fit (PPP = 0.006). This suggested that separate models for boys and girls were not appropriate. Thus, no support for significant differences in this model across gender groups was evident.

### Lagged Models, Sample 2

In the second sample, fit indices also indicated that the RI-CLPM had a better fit (PPP = 0.310) than the CLPM (PPP = 0.000). In this sample, four significant within-person lagged estimates were significant, all between depressive symptoms and the binary component of NSSI (see Table 3, right-hand columns). As shown in Fig. 1 (depicted as dashed lines), depressive symptoms at T1 predicted engaging in any NSSI at T2. This path was also found from T3 depressive symptoms to T4 NSSI. Finally, there were also lagged estimates from the binary component of NSSI to depressive symptoms, within the same lags as in the first, original sample (T2-T3 and T3-T4 lags). These lags can be interpreted as meaning that any engagement in NSSI predicted increases in depressive symptoms.

Two sets of lags were tested for equivalence. The equivalence test for the first set involved comparing the path from NSSI (binary) to depressive symptoms from T2 to T3 and from T3 to T4. The lags did not significantly differ in magnitude (Wald’s χ2 difference = 0.77, *df* = 1, *p* = 0.38). Differences in the cross-lags between T3 and T4 were also tested, from NSSI (binary) to depressive symptoms and vice-versa. These paths did not significantly differ in magnitude (Wald’s χ2 difference = 2.36, *df* = 1, *p* = 0.12).

As with the first sample, the model estimating paths separately for boys and girls did not fit the data well (PPP = 0.000) suggesting no support for gender differences.

## Discussion

Previous research examining the directionality of temporal links between NSSI and depressive symptoms during adolescence used analyses that did not control for between-person variance or account for the semi-continuous distribution of NSSI. Thus, findings from previous research needed to be replicated. To correct these analytical issues, two-part CLPMs were compared to two-part RI-CLPMs. The superior fit of the RI-CLPMs demonstrated that controlling for between-person variance with RI-CLPMs was needed to estimate the temporal ties between NSSI and depressive symptoms. Across both samples, the results showed a robust transactional pattern between depressive symptoms and NSSI. As expected, the transactional process began with depressive symptoms in early adolescence, which are then related to increases in NSSI. This is consistent with theory and research implicating strong, negative emotions in the onset of NSSI. Across the next lags, when most participants were moving into and through middle adolescence, the transactional pattern emerges. In the second sample, collected approximately 5 years after the first sample, a link was also found in the third lag, suggesting bidirectionality between depressive symptoms and NSSI. This may explain why NSSI and depressive symptoms are more closely tied after early adolescence. No evidence of ties were found in the last lag of the second sample, when most participants were in late adolescence.

A notable difference in results across samples in this study was the component of NSSI that was significantly tied to depressive symptoms. In the first sample collected, the ties were with the continuous element of NSSI, whereas the binary element was connected to depressive symptoms in the second sample. Although this may be due to sample fluctuations, it may also be that ties in the later cohort were strong enough that *any* NSSI had implications for depressive symptoms. Continuing to examine both binary and continuous elements of NSSI could provide meaningful information about its development.

The results of this study differ somewhat from others, where the most robust findings suggest more transactional processes than bidirectionality. The differences may be due, in part, to age differences across samples. However, the differences may also be attributed to bias introduced when between-person variance is uncontrolled. In this study, including the random intercepts was needed to maximize model fit to the data. In addition, two-part models accounted for the semi-continuous distribution of NSSI. Future studies need to account for these issues, directly testing, rather than assuming that trait-like stability is not affecting results.

An additional aim of the current study was to explore the potential effect of gender. Although no support for gender differences were found in this or other studies (e.g., Buelens et al., 2019), the relative lack of studies using RI-CLPMs means that gender differences cannot be ruled out. Caution is also needed when interpreting the non-significant lags as true nulls. Weak and non-significant associations can emerge when there is unidentified heterogeneity. Thus, future research should focus on conditions that could moderate the paths.

The current study has many strengths including its two large community samples, use of multi-wave longitudinal data and established measures, and the use of methods that account for both temporal and time-invariant, within-person stability. Despite these strengths, there are limitations. First, constructs of interest across both samples were assessed using single reporters, which sometimes introduces shared method variance. Post-hoc analyses indicates this was not a problem with these data (Harmon’s one-factor test showed revealed only 42% of the variance in both samples was explained by a single factor, with >50% indicating common method bias). Moreover, assessment of NSSI involved estimates of past 6-month frequency, but no information about other aspects of NSSI (e.g., reasons, severity) was available. Methods of assessment impact the documented prevalence and characterization of adolescent NSSI (Aspeqvist et al., 2024). Future research involving multiple and more detailed methods of assessment will be important to enhance confidence in these findings and add depth to our understanding of these transactional associations.

Additionally, to better support the potential developmental progression of NSSI observed in the current study, data from younger adolescents are needed for comparison. If future studies following youth from early to late adolescence also highlight mid-adolescence as a period in which bidirectional linkages between depressive symptoms and NSSI emerge, early adolescent prevention efforts to reduce comorbidity may be further justified.

Finally, as suggested by research on both depressive symptoms (Hankin & Abramson, 2001) and NSSI (Selby et al., 2008) a tendency toward repetitive, negative thinking (i.e., rumination) may strengthen ties between these two problem areas. Future research may be well served by exploring potential transdiagnostic factors, such as rumination or other maladaptive emotion regulation strategies, that may serve mechanistic functions in establishing and maintaining these transactional associations over time.

## Conclusions

Given the frequency of co-occurrence between NSSI and depressive symptoms during adolescence, researchers have aimed to understand the temporal ties between these two psychological problems. Despite longitudinal samples, such understanding was limited by the use of methods that could bias results. Such methodological shortcomings included applying analyses that did not account for the semi-continuous distribution of NSSI and neglecting to test if there was trait-like continuity in either NSSI or depressive symptoms that needed to be controlled. Using RI-CLPM and two-part methods, these results suggest that from early to late adolescence, the links between NSSI and depressive symptoms were at transactional, rooted in depressive symptoms during early adolescence. In addition, in at least one sample, evidence emerged that suggested bidirectionality during middle adolescence. Such findings suggest the potential for a maintenance cycle of comorbid distress. Our findings suggest that interventions aimed at preventing or reducing depression in early adolescence, before age 15, may be optimal for reducing the co-occurrence of depressive symptoms and NSSI, interrupting transactional processes before they begin. Such efforts could focus on the development of adaptive strategies for managing emotional distress, helping to reduce both NSSI and depressive symptoms during this formative developmental period.

## Supplementary information

Supplemental Files
