# Supplementary material for: Non-Suicidal Self-Injury and Depressive Symptoms During Adolescence: Testing Directionality
Source: J Youth Adolesc. 2025 Apr 13;54(9):2168–79. doi: 10.1007/s10964-025-02183-y (PMC12420763; doi:10.1007/s10964-025-02183-y)
Supplement: Supplementary file 1 — Supplemental Files [file 10964_2025_2183_MOESM1_ESM.docx]

Supplemental Files

**Table 1**

*Previous Research on NSSI and self-harm: Sample, Measurement, and Design Characteristics, and Results*

|  |  | NSSI measure | Sample age at T1 | | Lags | | 1^st^ lag | | 2^nd^ lag | |  |
| --- | --- | --- | --- | --- | --- | --- | --- | --- | --- | --- | --- |
|  | *N* |  | Range | *M* years  (*SD*) | # | Duration (months) | NSSI to DS/PD | DS/PD to NSSI | NSSI to DS/PD | DS/PD to NSSI | Models used |
| Hu et al., 2023 | 6,023 | 1 binary item, lifetime | 10-17 | 11.63 (1.54) | 1 | 7 | .02** | .26** | - | - | CLPM |
| Lei et al., 2024 | 1,143 | scale,  lifetime | 11-15 | 13.10 (0.93) | 2 | 6 | .06* | .13** | .06** | .13** | CLPM |
| Marshall et al., 2013 | 506 | scale  6 months prior | 12-14 | 13.21 (0.57) | 2 | 12 | .09 | .21** | .01 | .07 | CLPM |
| Buelens et al., 2019 | 526 | 1 binary item, lifetime | 11-19 | 15.00 (1.85) | 1 | 12 | .12** | .23** | .16** | .19** | CLPM |
| Faura-Garcia et al., 2023 | 780 | 1 ordinal item,  1 year prior | 13-18 | 15.64 (1.08) | 1 | 12 | .20* | .15** | - | - | CLPM |
| Garisch & Wilson, 2015 | 830 | scale, lifetime | 11-19 | 16.23 (0.56) | 1 | 5 | .08 | .12* | - | - | CLPM |

*Note.* NSSI = non-suicidal self-injury; DS/PD = depressive symptoms or internalized distress (in Buelens et al., 2019). Bolding indicates stronger path.

**p* < .05. ***p* < .01.

**Table 2**

*Results From Previous Research and This Study*

|  | Lag frame | | | | | |
| --- | --- | --- | --- | --- | --- | --- |
| Study | 11-12 | 12-13 | 13-14 | 14-15 | 15-16 | 16-17 |
| Hu et al. | bidirectional |  |  |  |  |  |
| Lie et al. |  |  | bidirectional |  |  |  |
| Marshall et al. |  |  | DS🡪NSSI | NS |  |  |
| Buelens et al. |  |  |  |  | bidirectional | bidirectional |
| Faura-Garcia et al. |  |  |  |  |  | bidirectional |
| Garisch & Wilson |  |  |  |  |  | DS🡪NSSI |
|  |  |  |  |  |  |  |
| Sample 1 (continuous) |  |  | DS🡪NSSI | NSSI🡪DS | DS🡪NSSI |  |
| Sample 2 (binary) |  |  | DS🡪NSSI | NSSI🡪DS | bidirectional | NS |

*Note.* Lag time frames are based on average age of samples and lag durations. NSSI = non-suicidal self-injury; DS = depressive symptoms; NS = non-significant lag estimates.

**Supplemental Appendix 1:**

**Commands For 2-Part CLPM, RI-CLPM, and Multigroup RCLPM**

1. **2-Part CLPM**

TITLE: 2 part CLPM

DATA: FILE = XXX.dat !identifies data file;

DATA TWOPART:

NAMES = NSSIt1-NSSIt4; ! original symptoms variable; tx as cont

BINARY = SIbin1-SIbin4; !NSSI binary (0 - none; 1 = any);

CONTINUOUS = SIcon1-SIcon4; !NSSI positive continuous (0 recoded to missing);

CUTPOINT = 0;

TRANSFORM = none;

VARIABLE:

NAMES =

DSt1 DSt2 DSt3 DSt4

NSSIt1 NSSIt2 NSSIt3 NSSIT4;

USEVARIABLES =

DSt1-DSt4

SIbin1-SIbin4

SIcon1-SIcon4; !Note that variables created by DATA TWOPART must be listed;

CATEGORICAL = SIbin1-SIbin4; !Identifies binary variable as categorical;

MISSING = all(9999);

ANALYSIS:

ESTIMATOR = BAYES;

BITERATIONS = (10000);

! FBITERATIONS = 200;

THIN = 10;

PROCESSORS = 4; !Use with multiple processors to speed estimation;

ALGORITHM = GIBBS(RW);

MODEL:

! univariates;

DSt2^-DSt4^ PON DSt1^-DSt3^;

!DSt2^-DSt3^ PON DSt1^-DSt2^;

SIbin2^-SIbin4^ PON SIbin1^-SIbin3^;

!SIbin2^-SIbin3^ PON SIbin1^-SIbin2^;

SIcon2^-SIcon4^ PON SIcon1^-SIcon3^;

!SIcon2^-SIcon3^ PON SIcon1^-SIcon2^;

! bivariates;

DSt2^-DSt4^ PON SIbin1^-SIbin3^;

DSt2^-DSt4^ PON SIcon1^-SIcon3^;

SIbin2^-SIbin4^ PON DSt1^-DSt3^;

SIcon2^-SIcon4^ PON DSt1^-DSt3^;

! covariances:

DSt1^-DSt4^ PWITH SIbin1^-SIbin4^;

DSt1^-DSt4^ PWITH SIcon1^-SIcon4^;

OUTPUT: STANDARDIZED RESIDUAL TECH8 TECH10;

PLOT: TYPE = PLOT3;

1. **2-Part RI-CLPM**

TITLE: 2 part RI-CLPM

DATA:

FILE = XXXX.dat; *!names data file;*

DATA TWOPART: *! Commands used to recode the semi-continuous variable into two parts;*

NAMES = NSSIt1-NSSIt5; *!original variable name;*

BINARY = SIbin1-SIbin5; *! naming the binary variables (0 = no NSSI; 1 = any);*

CONTINUOUS = SIcon1-SIcon5; !*NSSI continuous (0 is missing; all other values retained);*

CUTPOINT = 0; *! identifies 0 as the cutpoint for binary categorization;*

TRANSFORM = none;

*! Standard variable information*

*! Note that the variables used from the dat file are the original variables;*

*! Variables created with data commands must be listed after named variables, in order;*

VARIABLE:

NAMES = gen

DSt1 DSt2 DSt3 DSt4 DSt5

NSSIt1 NSSIt2 NSSIt3 NSSIT4 NSSIt5;

USEVARIABLES =

DSt1-DSt5

SIbin1-SIbin5

SIcon1-SIcon5;

CATEGORICAL = SIbin1-SIbin5; *! Identifies the binary categorical variables;*

MISSING = all(9999);

ANALYSIS:

ESTIMATOR = BAYES;

BITERATIONS = (10000);

! FBITERATIONS = 200;

THIN = 10;

PROCESSORS = 4; ! use with multiple processors to speed calculations;

ALGORITHM = GIBBS(RW);

MODEL:

!random intercepts;

iDS BY DSt1-DSt5@1;

iSIb BY SIbin1-SIbin5@1;

iSIc BY SIcon1-SIcon5@1;

! univariates, analogous to auto-regressive estimates;

DSt2^-DSt5^ PON DSt1^-DSt4^;

DSt2^-DSt4^ PON DSt1^-DSt3^;

SIbin2^-SIbin5^ PON SIbin1^-SIbin4^;

SIbin2^-SIbin4^ PON SIbin1^-SIbin3^;

SIcon2^-SIcon5^ PON SIcon1^-SIcon4^;

SIcon2^-SIcon4^ PON SIcon1^-SIcon3^;

! bivariates, analogous to cross-lagged paths;

DSt2^-DSt5^ PON SIbin1^-SIbin4^;

DSt2^-DSt5^ PON SIcon1^-SIcon4^;

SIbin2^-SIbin5^ PON DSt1^-DSt4^;

SIcon2^-SIcon5^ PON DSt1^-DSt4^;

! covariances:

DSt1^-DSt5^ PWITH SIbin1^-SIbin5^;

DSt1^-DSt5^ PWITH SIcon1^-SIcon5^;

OUTPUT: STANDARDIZED RESIDUAL TECH8 TECH10;

PLOT: TYPE = PLOT3;

1. **Multigroup 2-Part RI-CLPM**

TITLE: 2 part RI-CLPM Multigroup to test for gender moderation

DATA: FILE = xxx.data;

DATA TWOPART:

NAMES = NSSIt1-NSSIt4; ! original symptoms variable; tx as cont

BINARY = SIbin1-SIbin4; !NSSI binary (0 - none; 1 = any);

CONTINUOUS = SIcon1-SIcon4; !NSSI positive continuous (0 recoded to missing);

CUTPOINT = 0;

TRANSFORM = none;

VARIABLE:

NAMES =

DSt1 DSt2 DSt3 DSt4

NSSIt1 NSSIt2 NSSIt3 NSSIT4;

USEVARIABLES =

DSt1-DSt4

SIbin1-SIbin4

SIcon1-SIcon4;

CATEGORICAL = SIbin1-SIbin4;

MISSING = all(9999);

Class = c(2);

KNOWNCLASS = c(gen = 1-2);

ANALYSIS:

TYPE = MIXTURE;

ESTIMATOR = BAYES;

BITERATIONS = (10000);

! FBITERATIONS = 200;

THIN = 10;

PROCESSORS = 4;

ALGORITHM = GIBBS(RW);

MODEL:

%OVERALL%

!random intercepts;

iDS BY DSt1-DSt4@1;

iSIb BY SIbin1-SIbin4@1;

iSIc BY SIcon1-SIcon4@1;

! univariates;

DSt2^-DSt4^ PON DSt1^-DSt3^;

DSt2^-DSt3^ PON DSt1^-DSt2^;

SIbin2^-SIbin4^ PON SIbin1^-SIbin3^;

SIbin2^-SIbin3^ PON SIbin1^-SIbin2^;

SIcon2^-SIcon4^ PON SIcon1^-SIcon3^;

SIcon2^-SIcon3^ PON SIcon1^-SIcon2^;

! bivariates;

DSt2^-DSt4^ PON SIbin1^-SIbin3^;

DSt2^-DSt4^ PON SIcon1^-SIcon3^;

SIbin2^-SIbin4^ PON DSt1^-DSt3^;

SIcon2^-SIcon4^ PON DSt1^-DSt3^;

! covariances:

DSt1^-DSt4^ PWITH SIbin1^-SIbin4^;

DSt1^-DSt4^ PWITH SIcon1^-SIcon4^;

%c#1%

[iDS@0 iSIb@0 iSIc@0];

%c#2%

[iDS iSIb iSIc];

OUTPUT: STANDARDIZED RESIDUAL TECH8 TECH10;

PLOT: TYPE = PLOT3;
